# Kidney-specific antigen depletion in human renal carcinomas.

**DOI:** 10.1038/bjc.1966.87

**Published:** 1966-12

**Authors:** R. C. Nairn, T. Ghose, A. E. Tannenberg

## Abstract

**Images:**


### KIDNEY-SPECIFIC ANTIGEN DEPLETION IN HUMAN RENAL CARCINOMAS

R. C. NAIRN, T. GHOSE AND A. E. G. TANNENBERG

*From the Department of Pathology, Monash University, Melbourne, Australia*

Received for publication August 8, 1966

DEPLETION of certain organ- and tissue-specific antigens has been reported in several human tumours including carcinoma of skin, cervix, colon, thyroid, testis and rhabdomyosarcomas, and in chemically induced hepatomas of the rat and the oestrogen-induced kidney carcinoma of the hamster (Weiler, 1956*a*, *b*, *c*, 1959 ; Nairn *et al.*, 1960, 1962*a* ; Hiramoto *et al.*, 1961*a*, *b*, 1962 ; Goudie and McCallum, 1962 ; Hillemanns, 1962). This communication presents evidence of depletion of kidney-specific antigen(s) in human renal carcinomas.

### METHODS

### *Antisera*

Three different antisera with kidney-specific activity were used. One anti-human kidney serum and an anti-hamster kidney serum had been produced in rabbits by immunization with microsomal material respectively from fresh surgically removed human kidneys or fresh hamster kidneys. They were the same sera as described in a previous study of kidney-specificity (Nairn *et al.*, 1962*b*). The third antiserum was obtained by immunizing neonatal rabbits with a homogenate of fresh human kidney (Tannenberg, 1967).

All the antisera when tested by immunofluorescence, gel diffusion or complement fixation, contained antibodies to tissue antigens other than kidney, and these were removed by successive absorptions with homogenates of human liver, lung and spleen ; the anti-hamster kidney serum was also absorbed with sheep erythrocytes to remove Forssman antibody and any anti-hamster species activity still remaining could be ignored for the present experiments. After absorption, the three sera showed specific antibody activity against human kidney and against the kidneys of all members of a wide range of other vertebrate animals examined (as yet unpublished).

### *Tumours*

Twelve kidney cancers and many more samples of normal kidney have been examined with generally concordant results but comprehensive investigation has been restricted to 5 fresh operation specimens from adults. These were selected because they each permitted adequate sampling of non-necrotic cancer and normal kidney both for immunohistology and serum absorptions. Any serological problems which might arise from individual-specific antigenicity could thereby be avoided. The tumours included three classical clear cell carcinomas, one similar tumour in which there were areas of papillary and tubular growth, and one well-differentiated transitional cell papillary carcinoma invading the kidney from the pelvis.

*Immunohistology*

Representative tissue blocks, about 4 × 4 × 2 mm., from the 5 specimens of kidney cancer were taken to provide samples consisting of (a) normal kidney, (b) cancer, and (c) kidney-cancer junction. They were snap-frozen in a liquid nitrogen-isopentane slurry at − 160° C. and stored at − 70° C. until required for sectioning. Frozen sections, 6 $\mu$ thick, were prepared in a cryostat at − 20° C. for immunofluorescent staining, which was carried out by the "sandwich" method with goat anti-rabbit-globulin labelled with fluorescein isothiocyanate or lissamine rhodamine B (RB 200). Specificity of staining was controlled by serological absorptions with appropriate normal tissue and cancer homogenates. The general immunofluorescence methods and tests of specificity employed are described in detail elsewhere (Nairn, 1964).

### RESULTS

The results are summarized in Table I, in which all three sera, having much the same reactivity, are considered together. The unabsorbed sera stained the kidneys and cancers diffusely, the former rather more intensely, and also sections

TABLE I.—*Immunofluorescent Staining by Anti-kidney Sera of Human Kidney and Kidney Cancers*

| Serum absorptions | Staining by 3 antisera (versus (1) human kidney homogenate, (2) human kidney microsomes, (3) hamster kidney microsomes) of: 5 normal kidneys | 4 renal carcinomas | 1 renal pelvic carcinoma |
|---|---|---|---|
| Nil | Diffuse ++++ | Diffuse +++ | Diffuse ++ |
| Human liver, lung, spleen (+ sheep erythrocytes for anti-hamster serum) | Proximal tubules +++ | Cancer cells + | − |
| | Distal ,, ++ | − | − |
| ,, + renal pelvic tumour | Glomeruli (+) | − | − |
| ,, + renal carcinoma (any of the 4) | Proximal tubules +++ | − | − |
| | Distal ,, + | − | − |
| | Glomeruli − | − | − |
| ,, + normal kidney | − | − | − |

(+) = weak, inconstant staining.

of several other human organs examined. The anti-hamster kidney serum, lacking human species antibodies, gave less staining that was not kidney-specific. By the absorptions with human tissues other than kidney or renal carcinoma, the sera were rendered kidney-specific and gave staining of non-neoplastic renal parenchyma which was practically confined to the tubules, being only weak and inconstant in the glomeruli (Fig. 1). These sera stained the renal cancer cells with moderate intensity (Fig. 2) but not the renal pelvic tumour nor other human organs. The same pattern and intensity of staining were obtained even after further serum absorptions with the renal pelvic tumour, indicating total lack of the kidney-specific antigens in this tissue.

When the sera were absorbed with homogenates of any of the 4 renal carcinomas, the pattern of staining they produced was changed. In the kidney, intense staining was limited to the proximal tubules, the distal nephron being relatively weakly stained and the glomeruli not at all (Fig. 3): there was no staining of the carcinomas (Fig. 4). Much the same results were obtained whether or not the sera were absorbed with the same carcinoma as used for staining, except that one of the clear cell carcinomas, itself staining relatively weakly, was a less effective absorbing material than the others: an additional absorption was required with this tumour to obtain complete inhibition of cancer staining. When sera that had been absorbed by tissues other than kidney were exposed to a single further absorption by normal kidney homogenate all staining was completly inhibited. In contrast, replacing the single kidney absorption by even as many as 4 equivalent absorptions by renal carcinoma homogenates did not prevent staining of normal kidney.

The kidney-specific staining was cytoplasmic in distribution, in some preparations showing greatest intensity at cell surfaces. In the renal carcinomas, staining was more conspicuous at cell surfaces and, in the clear cell tumours it had a speckled cytoplasmic pattern, while in the papillary tumour it had a more solid appearance. This presumably reflected the relative paucity of non-antigenic lipids and polysaccharides in the cytoplasm of the papillary growth.

### DISCUSSION

These results indicate that human renal carcinomas contain at least one kidney-specific antigen which is found in the kidney itself distributed throughout the nephron. On the other hand, the tumours lack at least one other kidney-specific antigen which in the normal kidney is confined to the tubules, being most abundant in the proximals. The antigen lack is certainly gross and may be total as judged by the ineffectiveness of multiple serum absorptions by the carcinomas in preventing kidney-specific staining. The complete absence of both antigens from the renal pelvic carcinoma is interesting in that it is in accord with conventional views on the histogenesis of this tumour. It also provided a valuable control experiment, supporting the validity of our findings with the other tumours. It is not possible from these observations to deduce the histogenesis of renal carcinomas, and there is nothing to support or deny the commonly accepted

---

### EXPLANATION OF PLATE

Unfixed frozen sections stained by the sandwich immunofluorescence technique with rabbit anti-human-kidney-microsome serum and goat anti-rabbit-globulin labelled with fluorescein isothiocyanate.

FIG. 1.—Normal part of human kidney treated with anti-kidney serum absorbed with human liver, lung and spleen. Bright staining of tubules. Glomeruli unstained in this preparation, the fluorescent specks being autofluorescent material only. × 250.

FIG. 2.—Clear cell renal carcinoma from same specimen and treated in the same way as in Fig. 1. Moderate surface and speckled cytoplasmic staining of cancer cells. × 250.

FIG. 3.—Same normal kidney treated as before except that the serum had been further absorbed by human renal carcinoma homogenate obtained from a different specimen. Bright staining of proximal convoluted tubules. Part of a distal tubule, bottom right, close to the glomerulus, with conspicuous non-fluorescent nuclei, is less intensely stained. The glomerulus shows autofluorescence only. × 250.

FIG. 4.—Same renal carcinoma treated as in Fig. 3. Complete absence of staining. Only autofluorescence visible. ×250.

1

2

3

4

Nairn, Ghose and Tannenberg.

view that they derive from the proximal tubular epithelium. The specific antigen they share with normal kidney is found distributed throughout the nephron.

The present additional evidence of loss of organ-specificity in spontaneous human cancer in conjunction with previous similar observations already quoted in the introduction, suggests that we are dealing with a general property of malignancy. Its relevance to tumour cell behaviour can only be a matter of speculation at the present time. The location of the normal kidney-specific antigen at the cell surface might suggest that it plays a role in cellular homeostasis, so that its absence could lead to uncontrolled growth. Furthermore, it is not unreasonable to suppose that loss of such a cell surface antigen might be associated with impairment of the normal control which restricts cells to their organ of origin, favouring tumour spread, and with a corresponding increase in capacity to colonize organs elsewhere.

### SUMMARY

Antisera prepared by injecting rabbits with kidney material were made kidney-specific by absorptions with homogenates of human organs other than kidney. They then reacted, as judged by immunofluorescent staining, strongly with normal human kidney and less with renal carcinoma. When the sera were further absorbed with renal carcinomas, only the capacity for staining non-neoplastic renal tubules remained. The findings indicate depletion of at least one kidney-specific antigen in human renal carcinomas. It is suggested that loss of organ-specificity may be a general feature of malignant change.

We wish to thank Mr. H. F. R. Story, Mr. J. S. Peters and other surgical colleagues for providing the operation specimens of kidney cancers, and Miss A. Hühn and Mr. A. Maxwell for technical assistance. The work was supported by grants from the Anti-Cancer Council of Victoria and the National Health and Medical Research Council.

### REFERENCES

GOUDIE, R. B. AND McCALLUM, H. M.—(1962) *Lancet*, i, 348.

HILLEMANNS, H. G.—(1962) *Z. Naturf.*, 17b, 240.

HIRAMOTO, R., BERNECKY, J., JURANDOWSKI, J. AND PRESSMAN, D.—(1961*a*) *Cancer Res.*, **21**, 1372.

HIRAMOTO, R., JURAND, J., BERNECKY, J. AND PRESSMAN, D.—(1962) *Proc. Soc. exp. Biol. Med.*, **111**, 505.

HIRAMOTO, R., JURANDOWSKI, J., BERNECKY, J. AND PRESSMAN, D.—(1961*b*) *Cancer Res.*, **21**, 383.

NAIRN, R. C.—(1964) ‘ Fluorescent Protein Tracing ’, 2nd edition. Edinburgh (Livingstone).

NAIRN, R. C., FOTHERGILL, J. E., McENTEGART, M. G. AND RICHMOND, H. G.—(1962*a*) *Br. med. J.*, i, 1791.

NAIRN, R. C., GHOSE, T., FOTHERGILL, J. E. AND McENTEGART, M. G.—(1962*b*) *Nature, Lond.*, **196**, 385.

NAIRN, R. C., RICHMOND, H. G., McENTEGART, M. G. AND FOTHERGILL, J. E.—(1960) *Br. med. J.*, ii, 1335.

TANNENBERG, A. E. G.—(1967) *Clin. exp. Immunol.*, **2**, in press.

WEILER, E.—(1956*a*) *Br. J. Cancer*, **10**, 553.—(1956*b*) *Br. J. Cancer*, **10**, 560.—(1956*c*) *Z. Naturf.*, 11b, 31.—(1959) ‘ Ciba Foundation Symposium on Carcinogenesis. Mechanisms of Action ’. London (Churchill), p. 165.


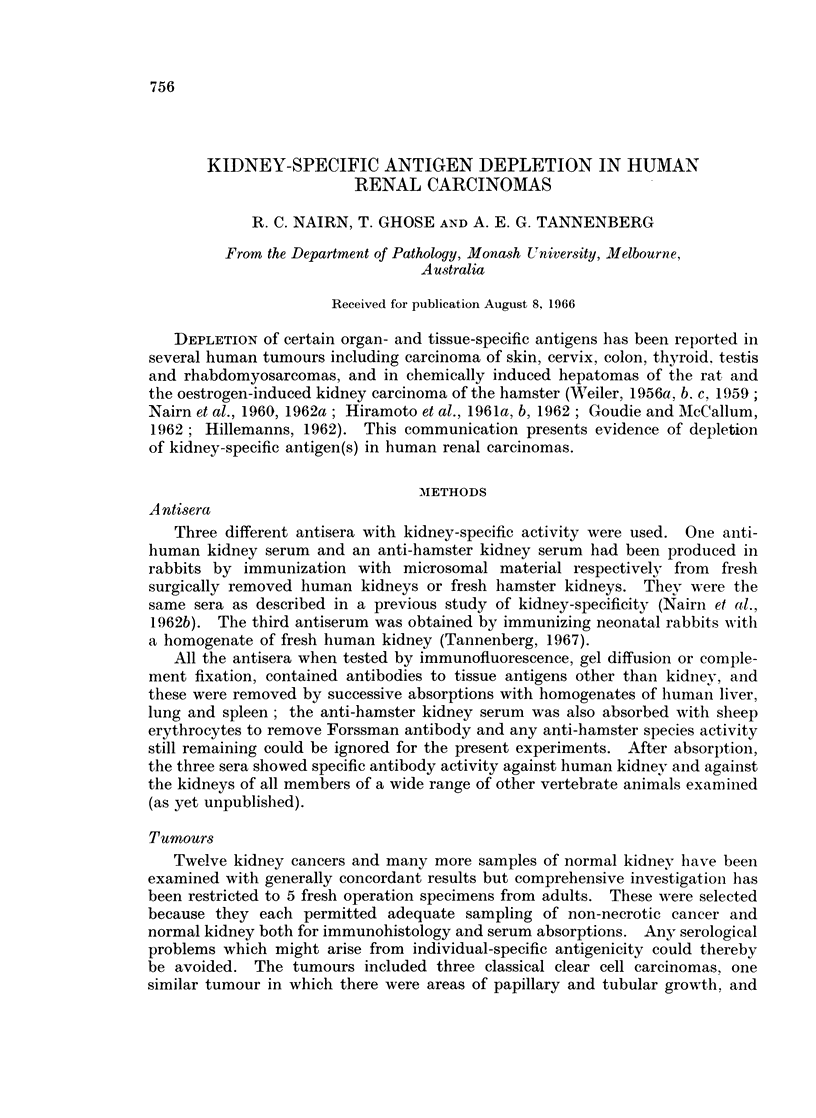

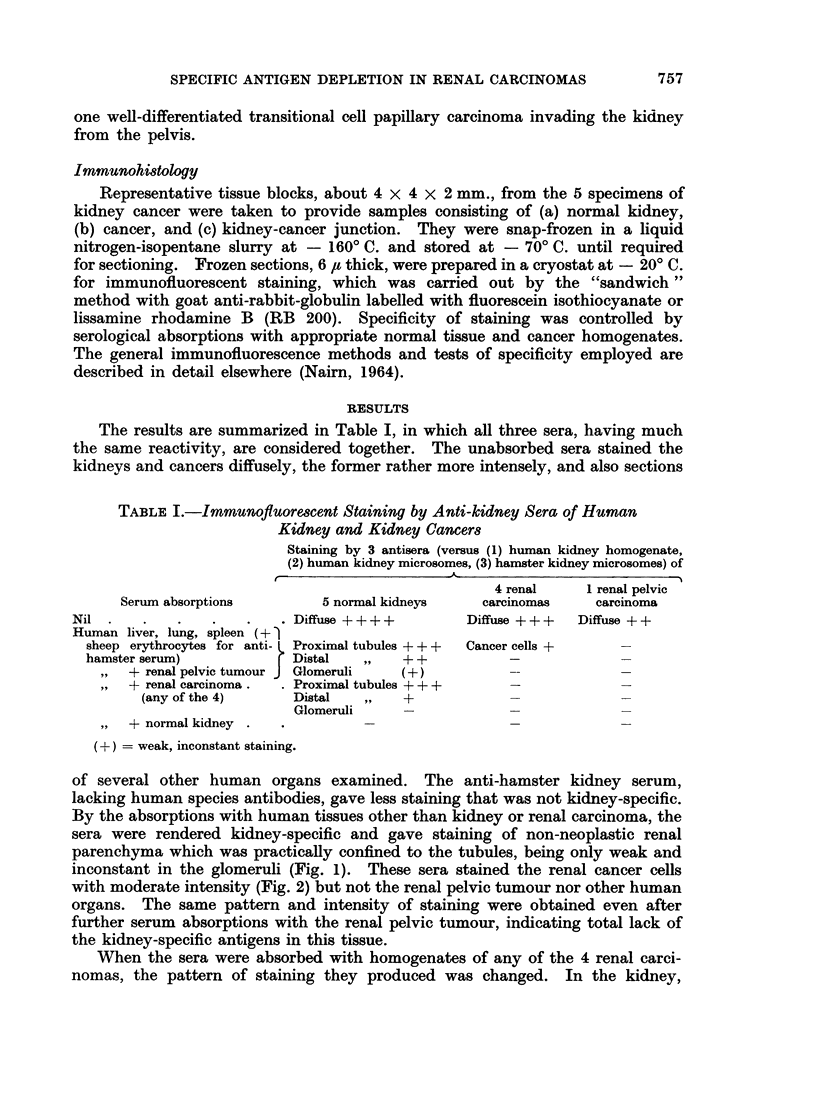

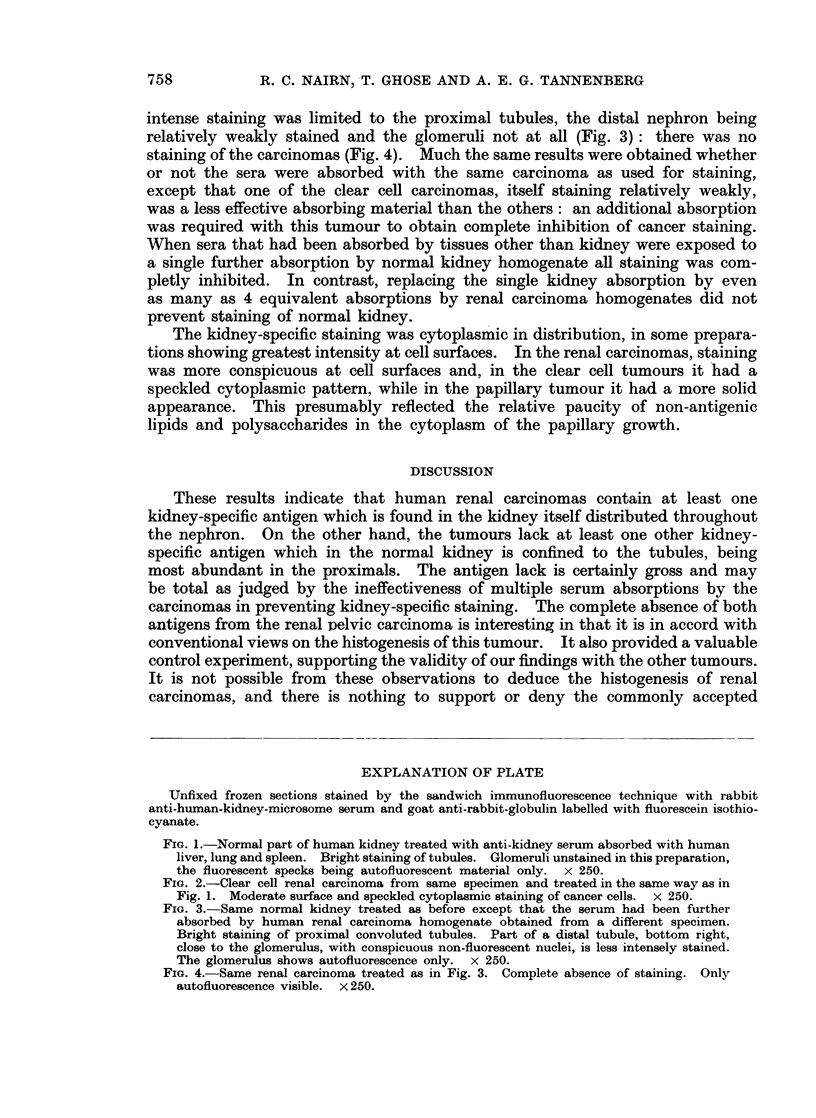

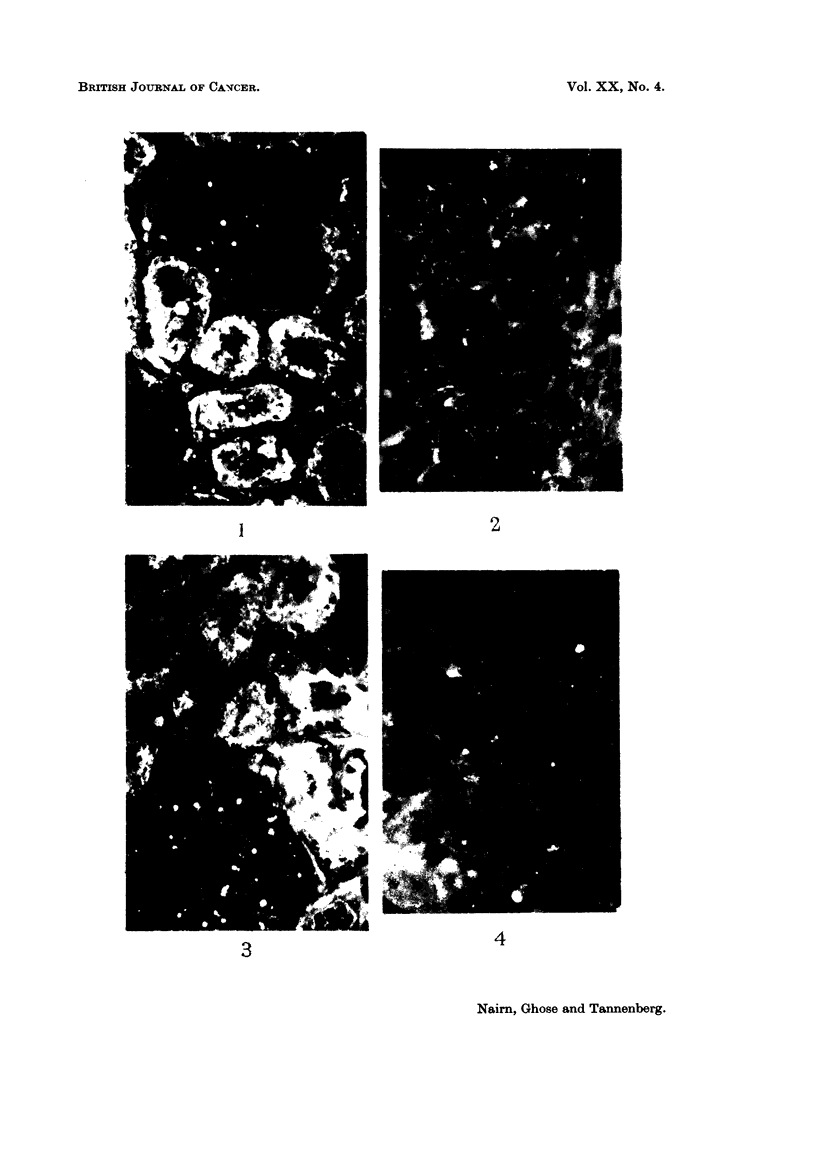

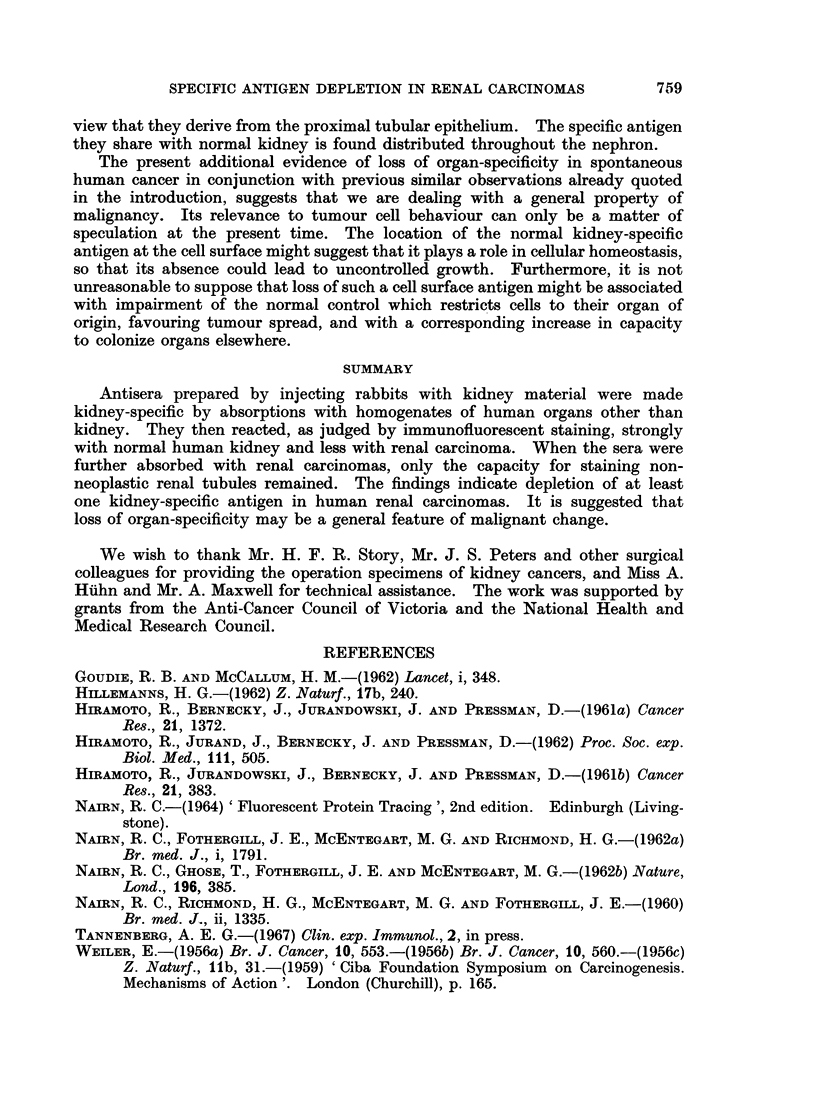


## References

[OCR_00237] HILLEMANNS H. G. (1962). [Serological and immunological studies on the pathogenesis of cervical cancer].. Z Naturforsch B.

[OCR_00239] HIRAMOTO R., BERNECKY J., JURANDOWSKI J., PRESSMAN D. (1961). Immunohistochemical staining properties of the N-2-FAA rat hepatoma.. Cancer Res.

[OCR_00243] HIRAMOTO R., JURAND J., BERNECKY J., PRESSMAN D. (1962). Lack of staining of testicular tumors by anti-sperm and anti-testis antibodies.. Proc Soc Exp Biol Med.

[OCR_00247] HIRAMOTO R., JURANDOWSKI J., BERNECKY J., PRESSMAN D. (1961). Immunochemical differentiation of rhabdomyosarcomas.. Cancer Res.

[OCR_00259] NAIRN R. C., GHOSE T., FOTHERGILL J. E., McENTEGART M. G. (1962). Kidney specific antigen and its species distribution.. Nature.

[OCR_00263] NAIRN R. C., RICHMOND H. G., McENTEGART M. G., FOTHERGILL J. E. (1960). Immunological differences between normal and malignant cells.. Br Med J.

